# Supplementary material for: Integrated in vivo combinatorial functional genomics and spatial transcriptomics of tumours to decode genotype-to-phenotype relationships
Source: Nat Biomed Eng. 2025 Jul 28;10(1):125–43. doi: 10.1038/s41551-025-01437-1 (PMC12823398; doi:10.1038/s41551-025-01437-1)
Supplement: Supplementary file 1 — Supplementary Figs. 1–18. [file 41551_2025_1437_MOESM1_ESM.pdf]

nature biomedical engineering

Supplementary information https://doi.org/10.1038/s41551-025-01437-1

Integrated in vivo combinatorial functional genomics and spatial transcriptomics of tumours to decode genotype-to-phenotype relationships

In the format provided by the authors and unedited

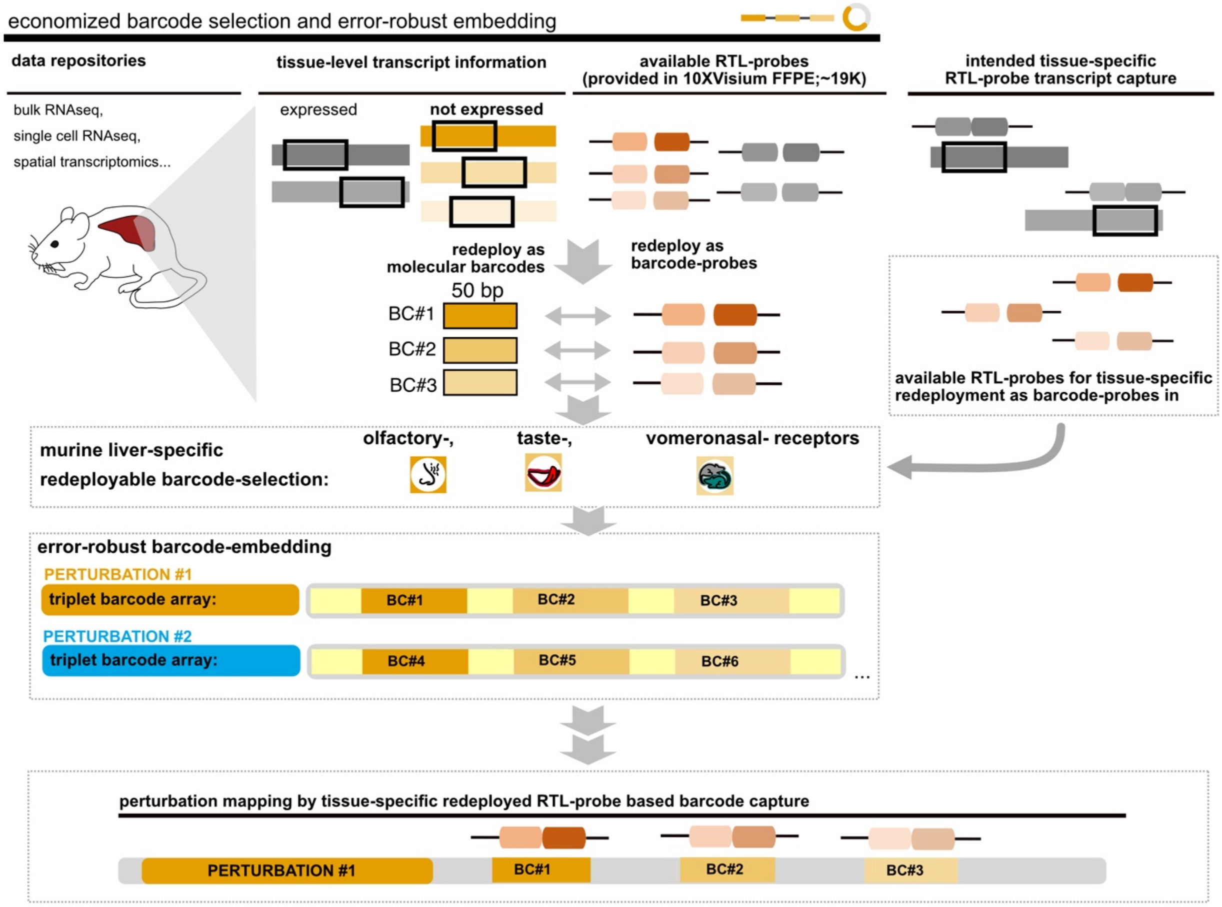

Supplementary Fig. 1: Economized barcode selection and error-robust embedding. Transcripts not expressed in tissue-of-interest (here murine liver) are identified using public databases. Their respective 50 nt RTL-probe capture sequences (available from 10X Genomics) are redeployed as detectable barcodes using commercially available RTL-probes against endogenous transcripts (provided with the 10X Visium for FFPE mouse kit; see Methods for details). Murine liver-specific redeployed barcodes are derived from olfactory-, taste-, and vomeronasal-receptor transcripts. Identified redeployable barcode sequences (Methods) are integrated into perturbation plasmids into triplet arrays. Consequently, three available RTL-probes enable identification of a single perturbation providing error-robustness. This means that if one barcode/RTL-probe pair proves non-functional, the presence of the other two sequences in the set ensure accurate identification of the perturbation. If two barcode/RTL-probe pairs prove non-functional, the presence of one sequence in the set ensures accurate identification of the perturbation. Only if three barcode/RTL-probe pairs prove non-functional, perturbation identification is unsuccessful.

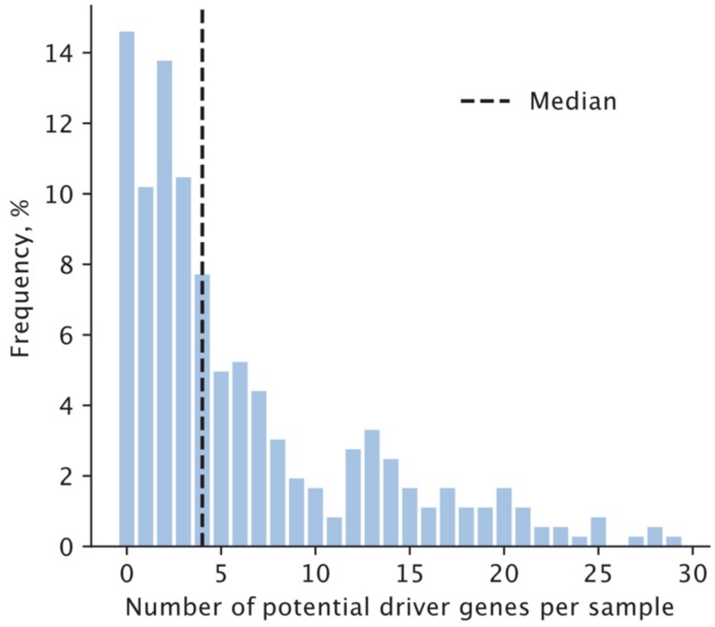

Supplementary Fig 2: Human liver tumors frequently present multiple genetic alterations. Frequency of potential driver mutations per sample in the TCGA-HCC dataset. Potential drivers were defined as either amplification or fusion of known COSMIC oncogenes, or homozygous deletion, nonsense mutation, splice site mutation, or frameshift deletion/insertion in tumor suppressor genes. The median value of 4 drivers per sample is indicated by the dashed line (data based on https://www.cbioportal.org/study/summary?id=lihc_tcga).

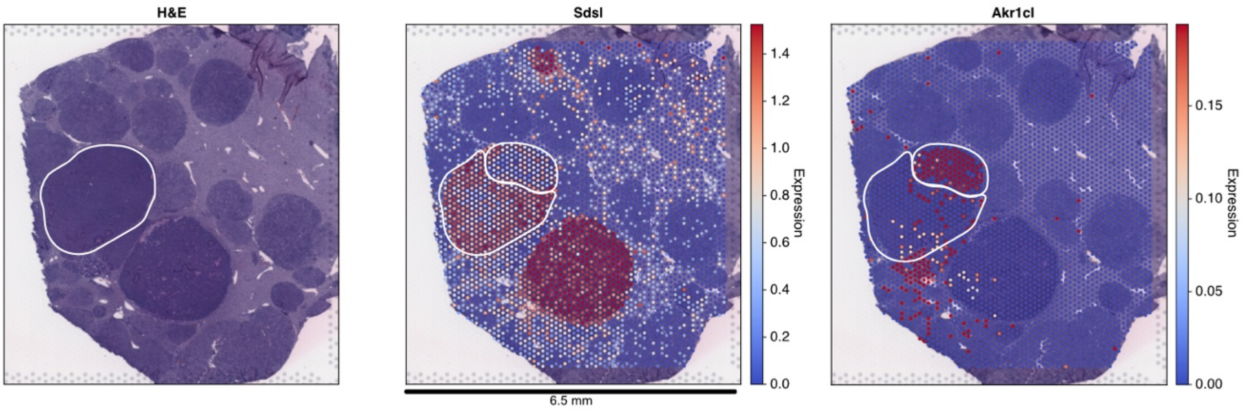

Supplementary Fig. 3: ST enables identification of overlapping nodules. H&E based annotation identified a single large tumor (left; encircled). ST differentiated this tumor into two nodules. To highlight example transcripts that enable differentiation, *Sdsl* is expressed in the larger tumor nodule, whereas *Akr1cl* is expressed in the smaller nodule. Representative example from a single 10X Visium ROI from a single RUBIX experiment with 2 animals.

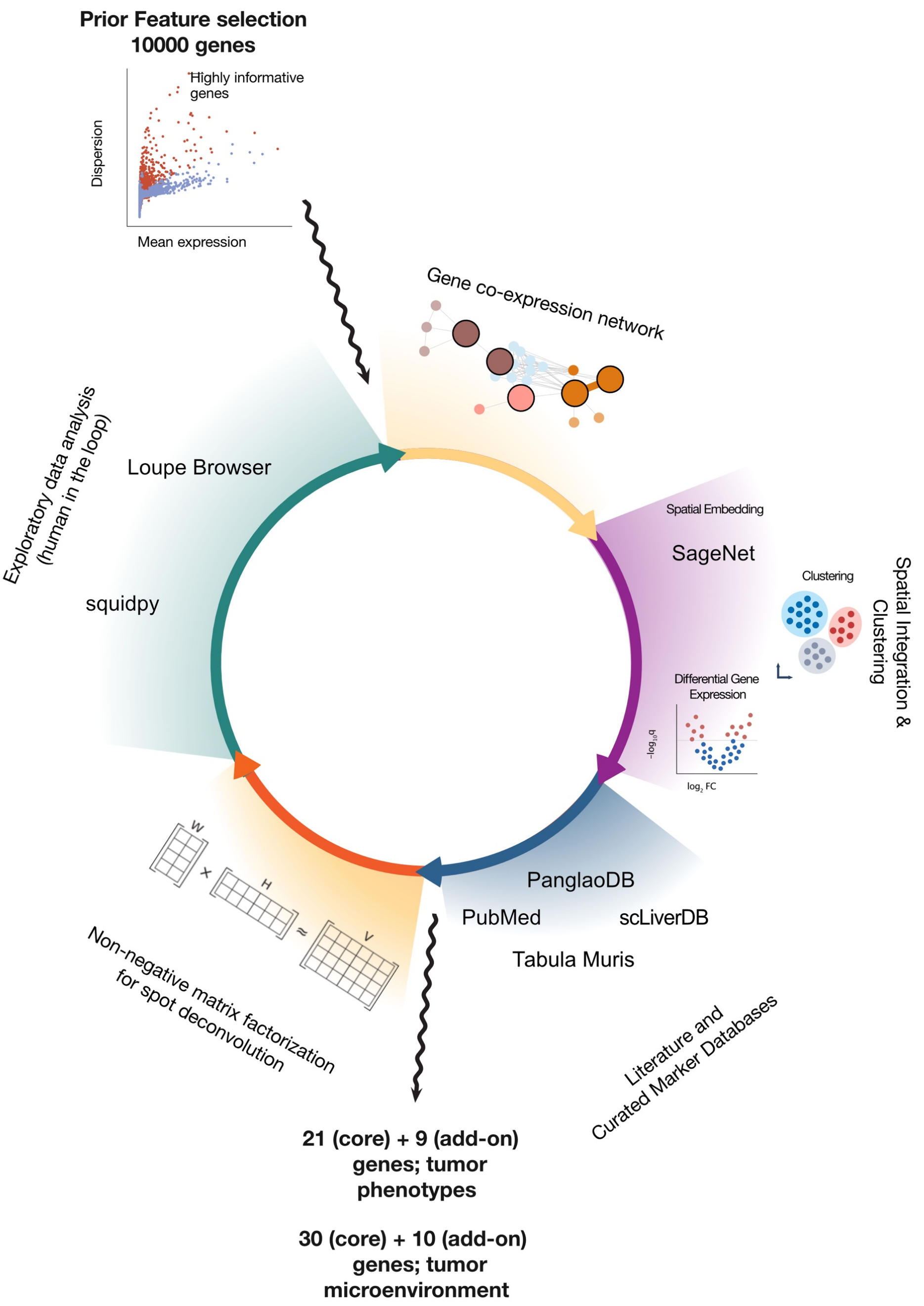

Supplementary Fig. 4: Isolating concise and informative sets of transcripts to characterize cell states. Beginning with a pool of 19,510 expressed genes across all ST samples, we identified the top 10,000 highly variable genes per sample, resulting in an overlap of 7,251 highly variable genes across 11 samples. Subsequently, we employed iterative processes involving hybrid data- and knowledge-driven approaches, along with manual verification of consistent gene expression patterns across samples and phenotypes. Through this process, we curated 21 “core marker” genes and 9 additional genes associated with tumor-intrinsic phenotypes, as well as 30 “core marker” genes and 10 additional genes linked to TME phenotypes.

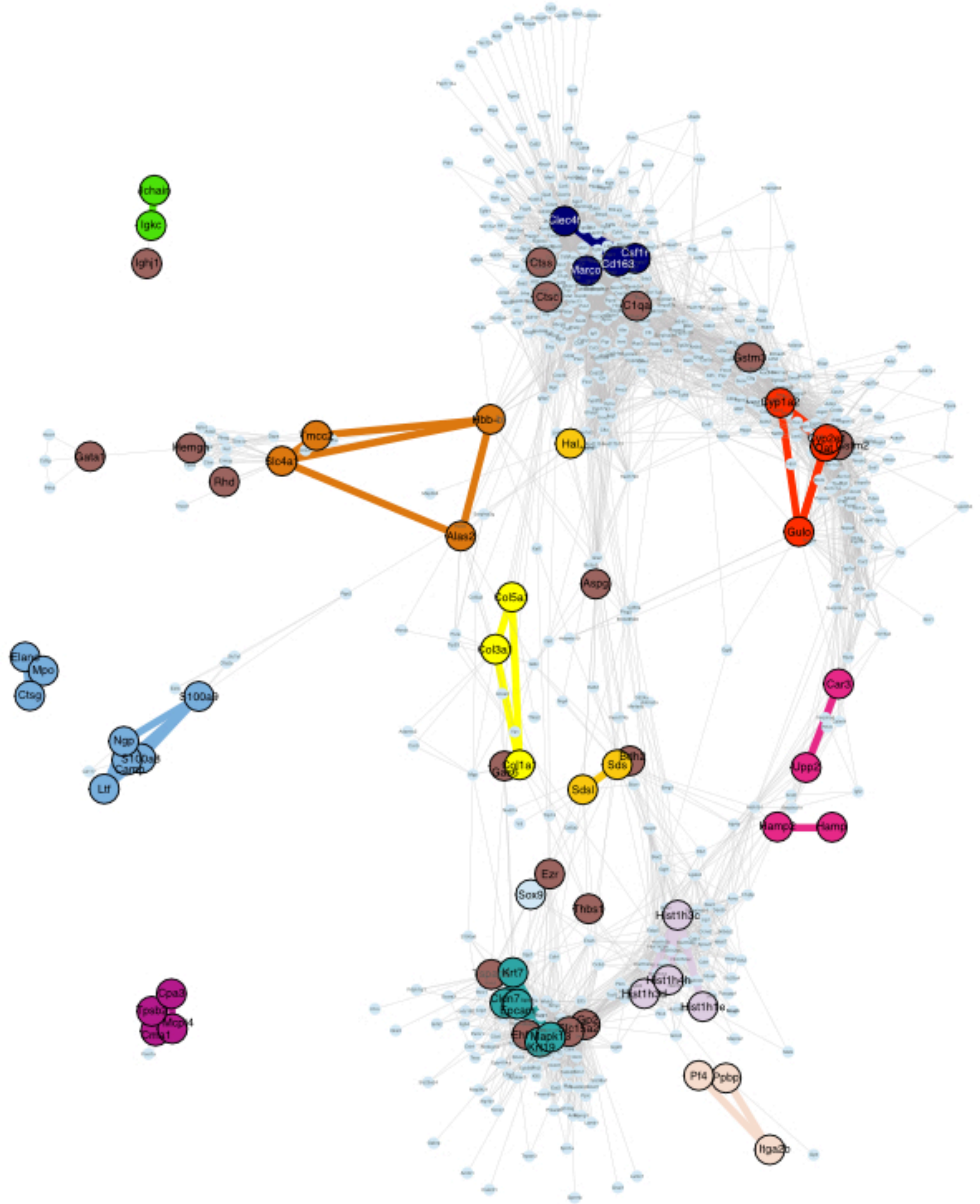

Supplementary Fig. 5: Transcript co-expression indicates phenotypic relationships and supports marker selection. Following the selection of phenotypic “core markers” and associated transcripts, we identified an intersection comprising 500 highly variable genes across all 11 samples from a single RUBIX experiment with 2 animals. Subsequently, we conducted a Gaussian graphical model analysis (Methods). Nodes correspond to genes, and edges signify relationships between pairs of genes. The node colors for “core markers” are indicative of their associated phenotype (see Fig. 5), while associated genes are depicted in brown. Edges connecting “core marker” genes of the same phenotype are emphasized. Isolated nodes lacking any assigned edges are excluded from the graph.

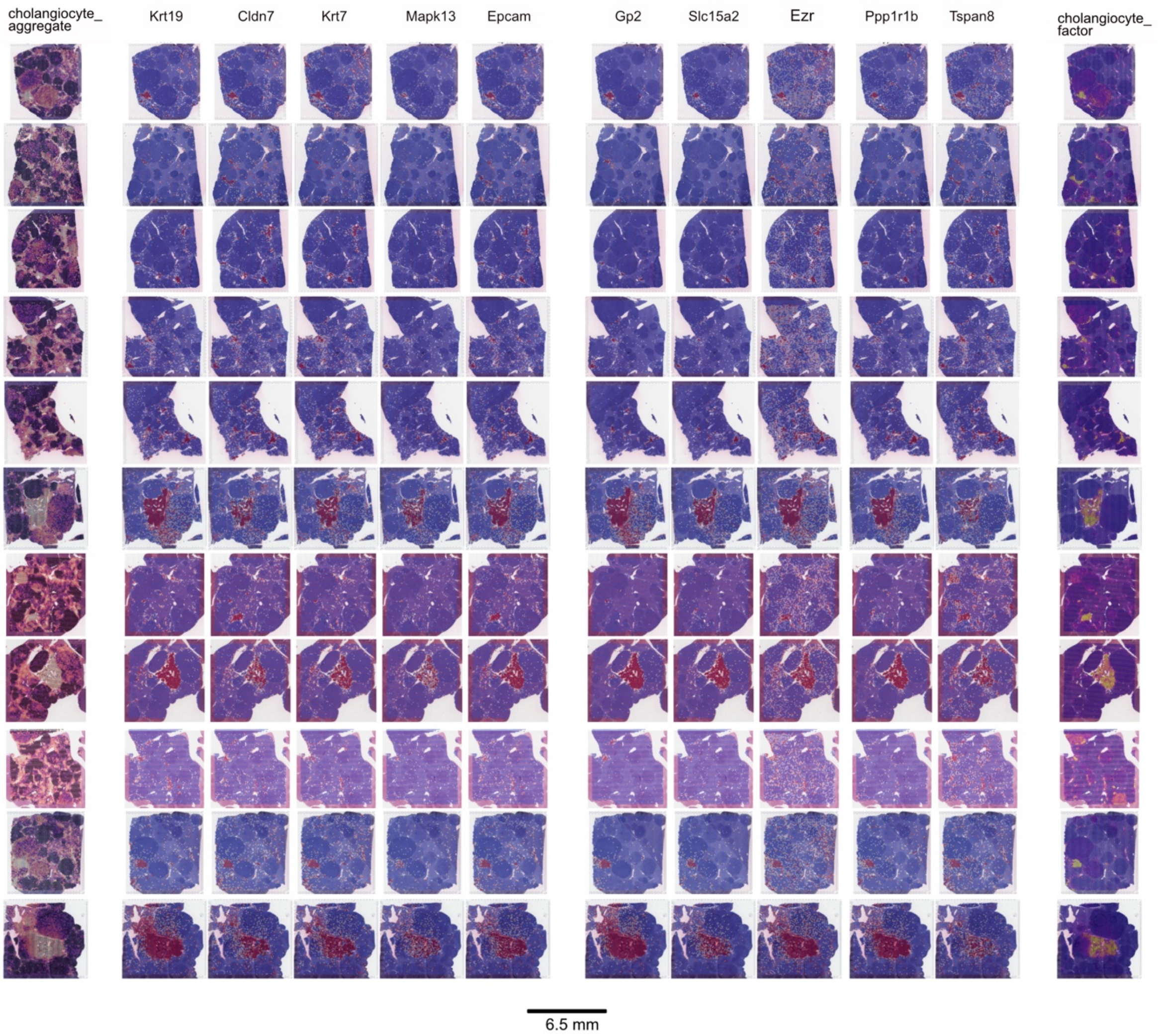

Supplementary Fig. 6: Cholangiocyte-like phenotype. Aggregated phenotypes, expression of individual phenotype-associated transcripts, and factor loadings per phenotype of interest. Left-most column: Aggregated phenotype values per each phenotype. Right-most column: Estimated factor loadings per spot (Methods). Columns in between: Scaled expression of single "core markers", as well as associated transcripts. As presented in Fig. 5. Data are depicted for all eleven 10X Visium samples used in this study. Samples are based on a single RUBIX experiment with 2 animals. See interactive web browser(https://chocolat-g2p.dkfz.de/).

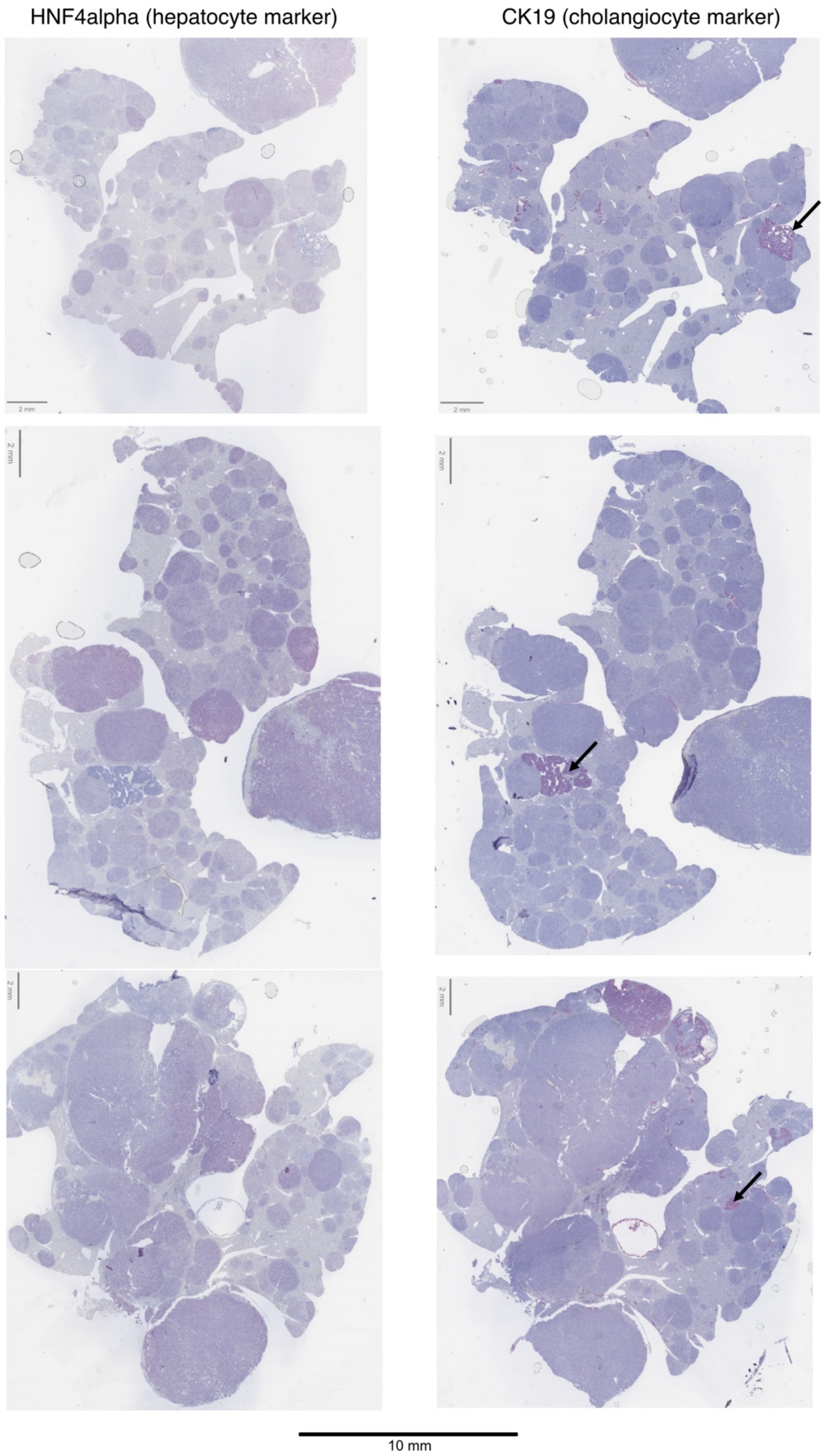

Supplementary Fig. 7: Immunohistochemistry-based identification of cholangiocytes/cholangiocarcinoma. Immunohistochemistry for HNF4a (hepatocyte marker) and CK19 (cholangiocyte marker). Arrows indicate prominent cholangiocarcinoma-nodules as an example. 3 whole-slide images from a single RUBIX experiments with 2 animals were used.

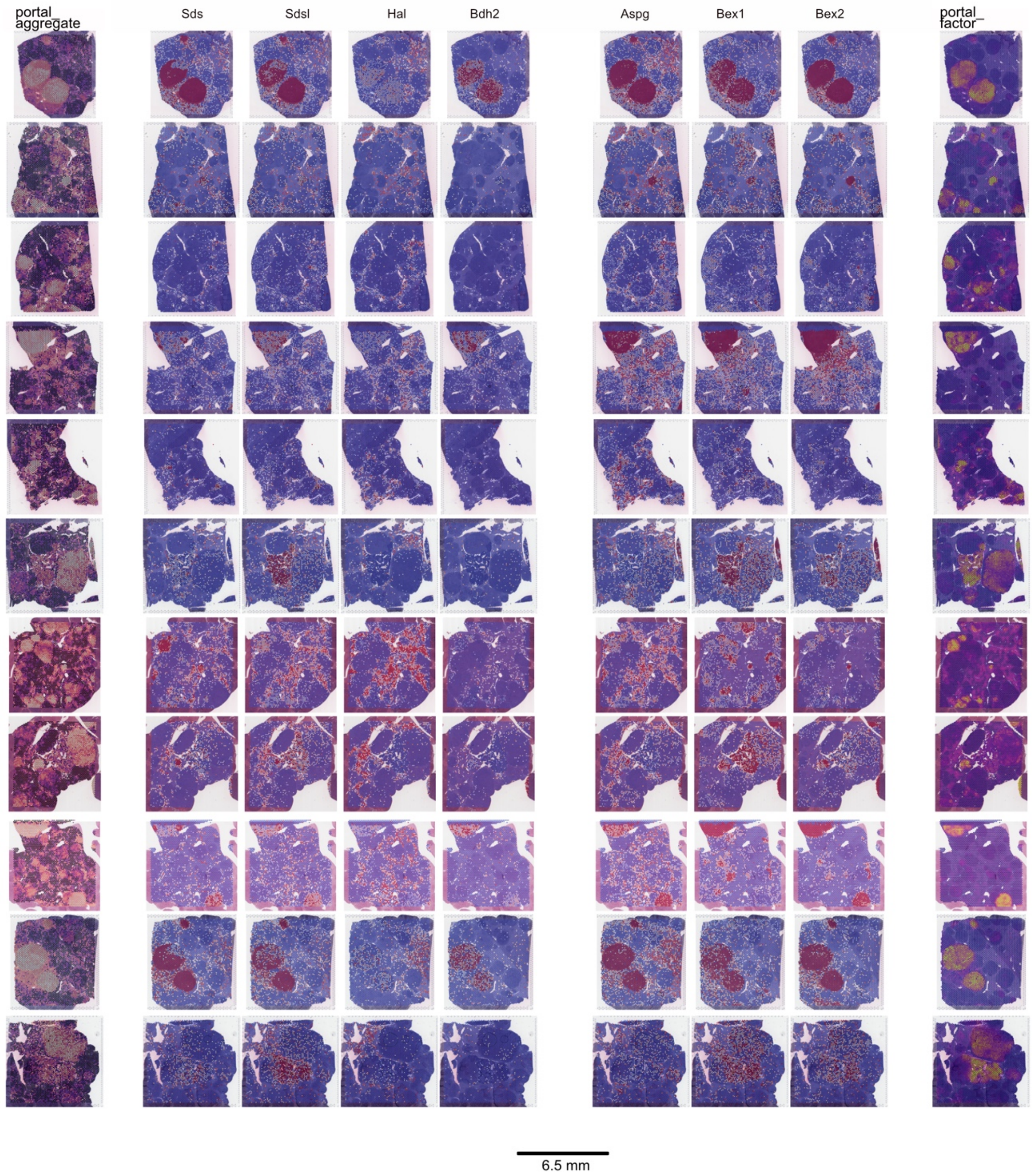

Supplementary Fig. 8: Portal-like phenotype. Aggregated phenotypes, expression of individual phenotype-associated transcripts, and factor loadings per phenotype of interest. Left-most column: Aggregated phenotype values per each phenotype. Right-most column: Estimated factor loadings per spot (Methods). Columns in between: Scaled expression of single "core markers", as well as associated transcripts. As presented in Fig. 5. Data are depicted for all eleven 10X Visium samples used in this study. Samples are based on a single RUBIX experiment with 2 animals. See interactive web browser(https://chocolat-g2p.dkfz.de/).

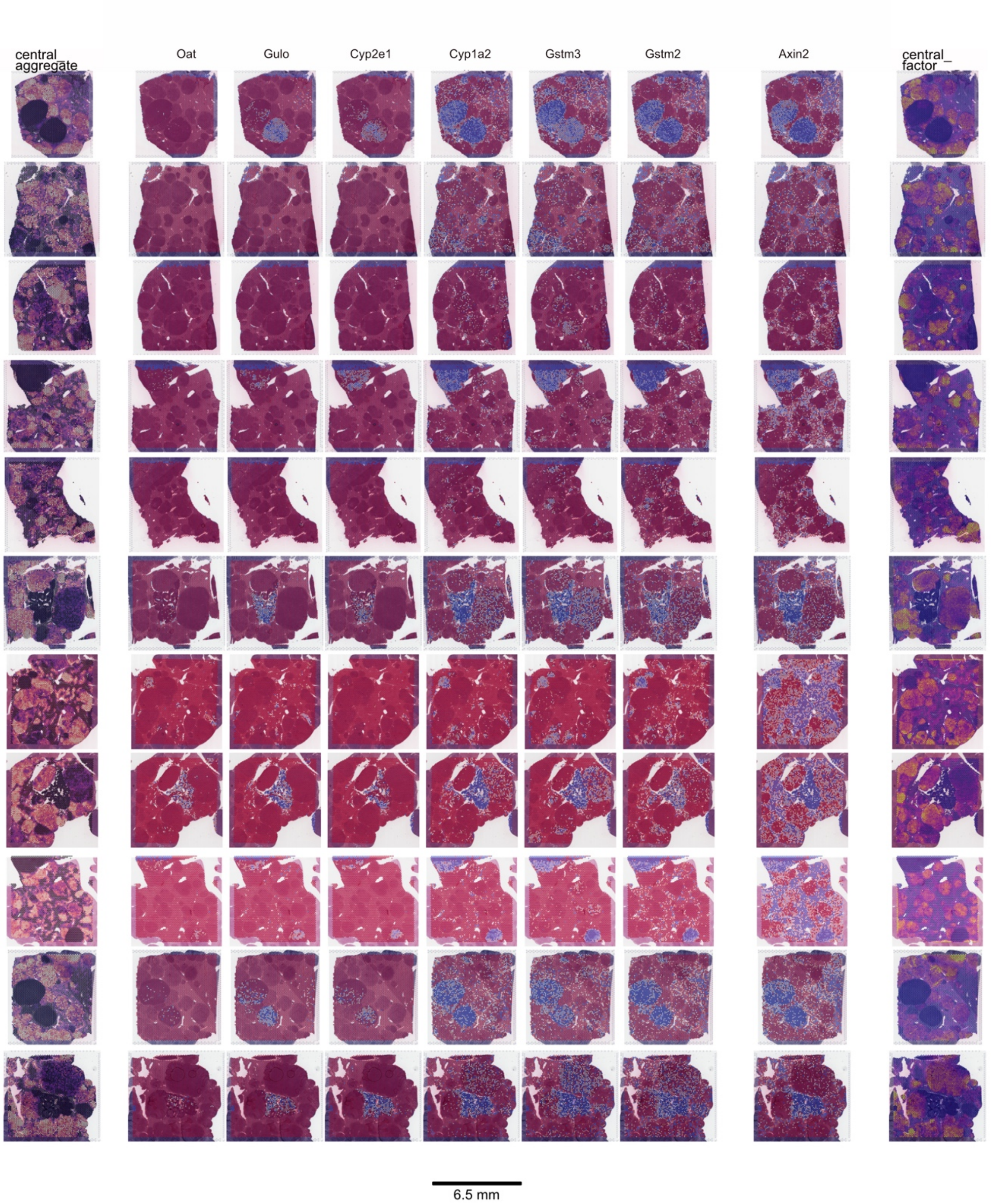

Supplementary Fig. 9: Central-like phenotype. Aggregated phenotypes, expression of individual phenotype-associated transcripts, and factor loadings per phenotype of interest. Left-most column: Aggregated phenotype values per each phenotype. Right-most column: Estimated factor loadings per spot (Methods). Columns in between: Scaled expression of single "core markers", as well as associated transcripts. As presented in Fig. 5. Data are depicted for all eleven 10X Visium samples used in this study. Samples are based on a single RUBIX experiment with 2 animals. See interactive web browser(https://chocolat-g2p.dkfz.de/).

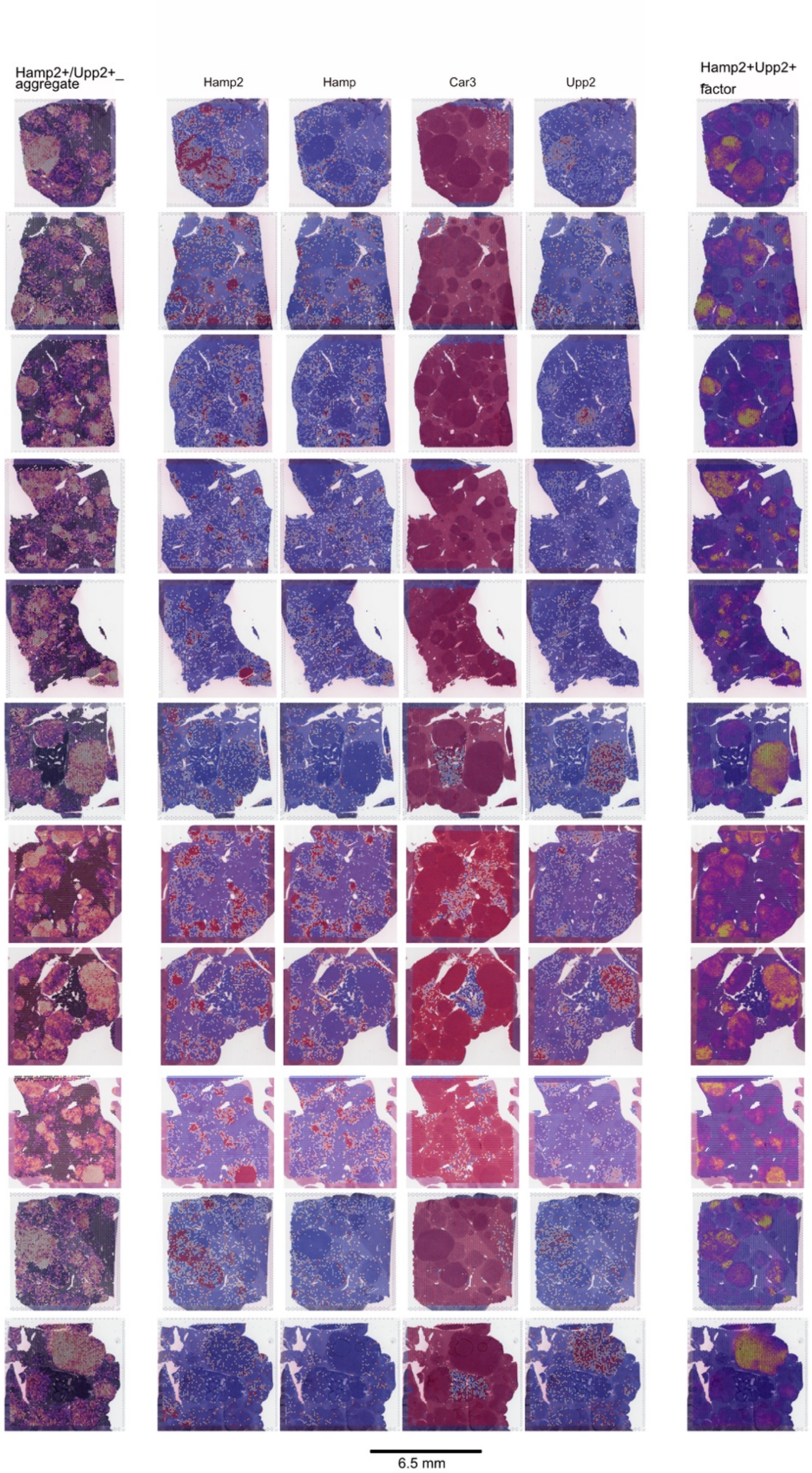

Supplementary Fig. 10: Hamp2+/Upp2+ phenotype. Aggregated phenotypes, expression of individual phenotype-associated transcripts, and factor loadings per phenotype of interest. Left-most column: Aggregated phenotype values per each phenotype. Right-most column: Estimated factor loadings per spot (Methods). Columns in between: Scaled expression of single "core markers", as well as associated transcripts. As presented in Fig. 5. Data are depicted for all eleven 10X Visium samples used in this study. Samples are based on a single RUBIX experiment with 2 animals. See interactive web browser(https://chocolat-g2p.dkfz.de/).

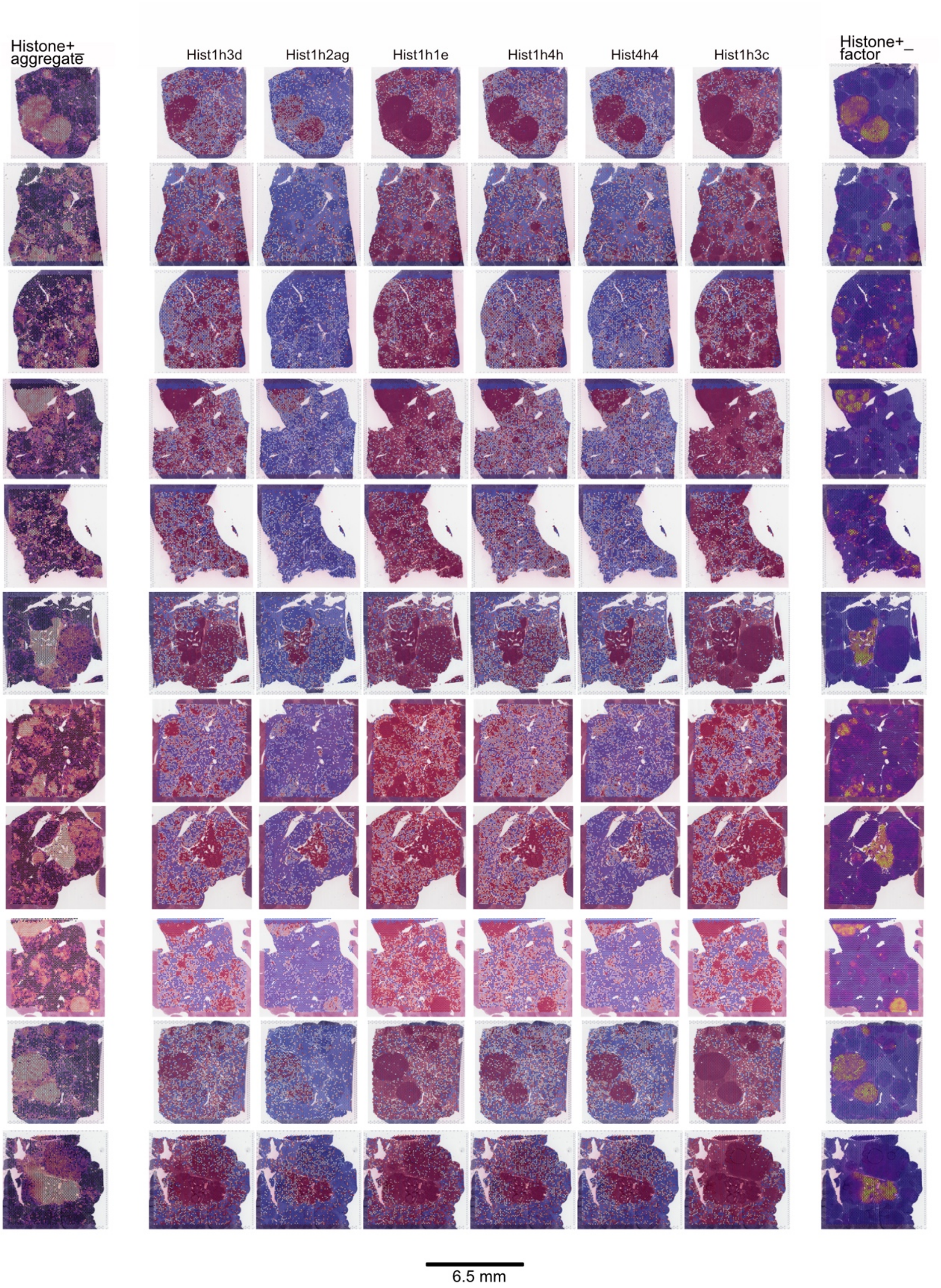

Supplementary Fig. 11: Histone+ phenotype. Aggregated phenotypes, expression of individual phenotype-associated transcripts, and factor loadings per phenotype of interest. Left-most column: Aggregated phenotype values per each phenotype. Right-most column: Estimated factor loadings per spot (Methods). Columns in between: Scaled expression of single "core markers", as well as associated transcripts. As presented in Fig. 5. Data are depicted for all eleven 10X Visium samples used in this study. Samples are based on a single RUBIX experiment with 2 animals. See interactive web browser(https://chocolat-g2p.dkfz.de/).

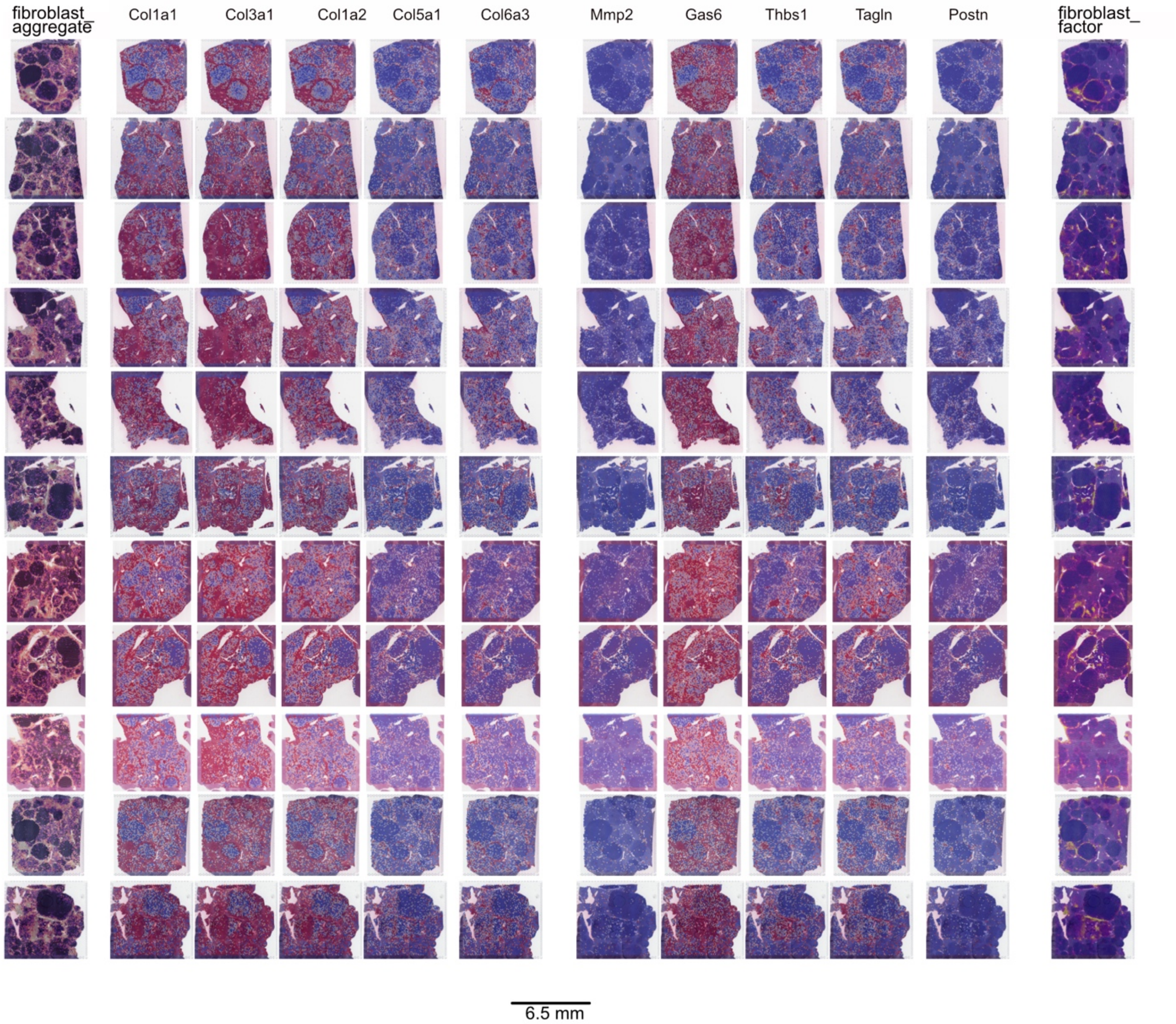

Supplementary Fig. 12: Fibroblast-like phenotype. Aggregated phenotypes, expression of individual phenotype-associated transcripts, and factor loadings per phenotype of interest. Left-most column: Aggregated phenotype values per each phenotype. Right-most column: Estimated factor loadings per spot (Methods). Columns in between: Scaled expression of single "core markers", as well as associated transcripts. As presented in Fig. 5. Data are depicted for all eleven 10X Visium samples used in this study. Samples are based on a single RUBIX experiment with 2 animals. See interactive web browser(https://chocolat-g2p.dkfz.de/).

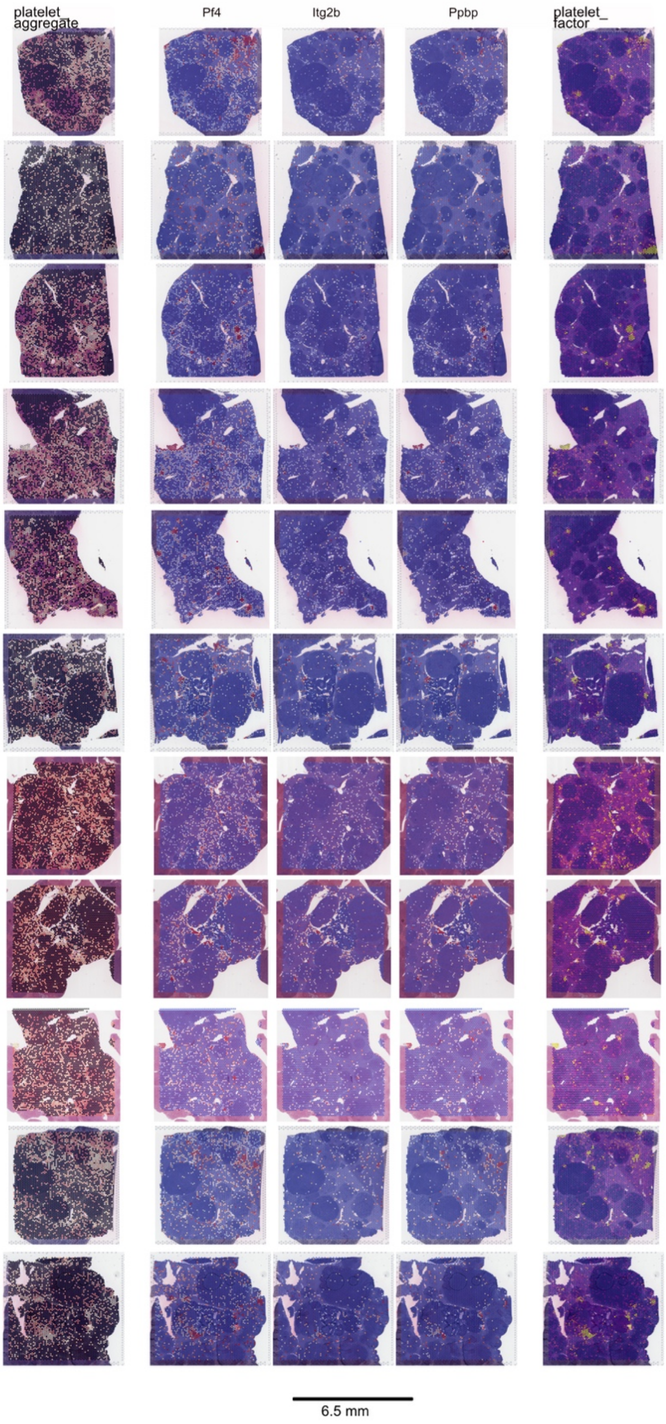

Supplementary Fig. 13: Platelet-like phenotype. Aggregated phenotypes, expression of individual phenotype-associated transcripts, and factor loadings per phenotype of interest. Left-most column: Aggregated phenotype values per each phenotype. Right-most column: Estimated factor loadings per spot (Methods). Columns in between: Scaled expression of single "core markers", as well as associated transcripts. As presented in Fig. 5. Data are depicted for all eleven 10X Visium samples used in this study. Samples are based on a single RUBIX experiment with 2 animals. See interactive web browser(https://chocolat-g2p.dkfz.de/).

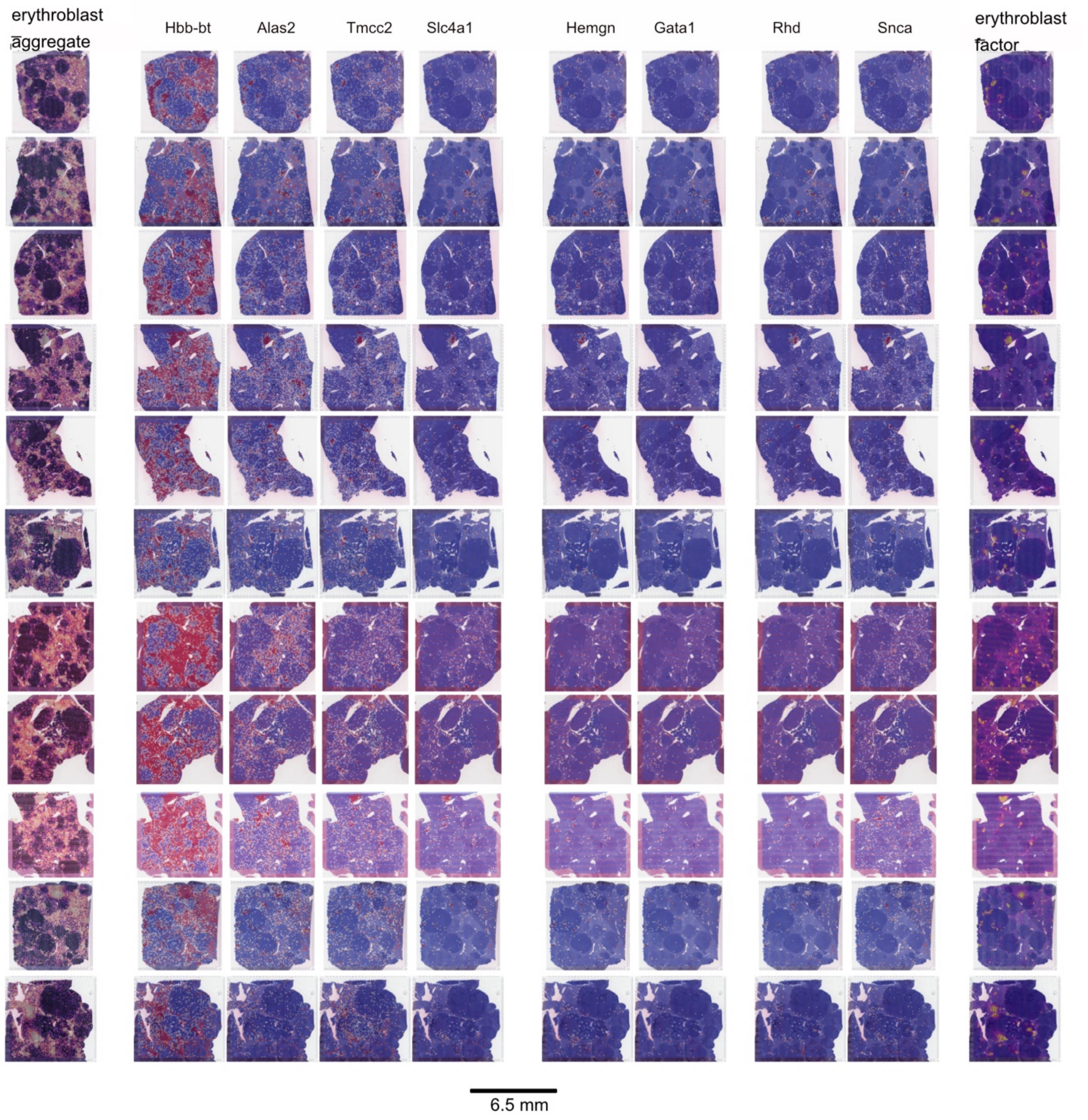

Supplementary Fig. 14: Erythroblast-like phenotype. Aggregated phenotypes, expression of individual phenotype-associated transcripts, and factor loadings per phenotype of interest. Left-most column: Aggregated phenotype values per each phenotype. Right-most column: Estimated factor loadings per spot (Methods). Columns in between: Scaled expression of single “core markers”, as well as associated transcripts. As presented in Fig. 5. Data are depicted for all eleven 10X Visium samples used in this study. Samples are based on a single RUBIX experiment with 2 animals. See interactive web browser(https://chocolat-g2p.dkfz.de/).

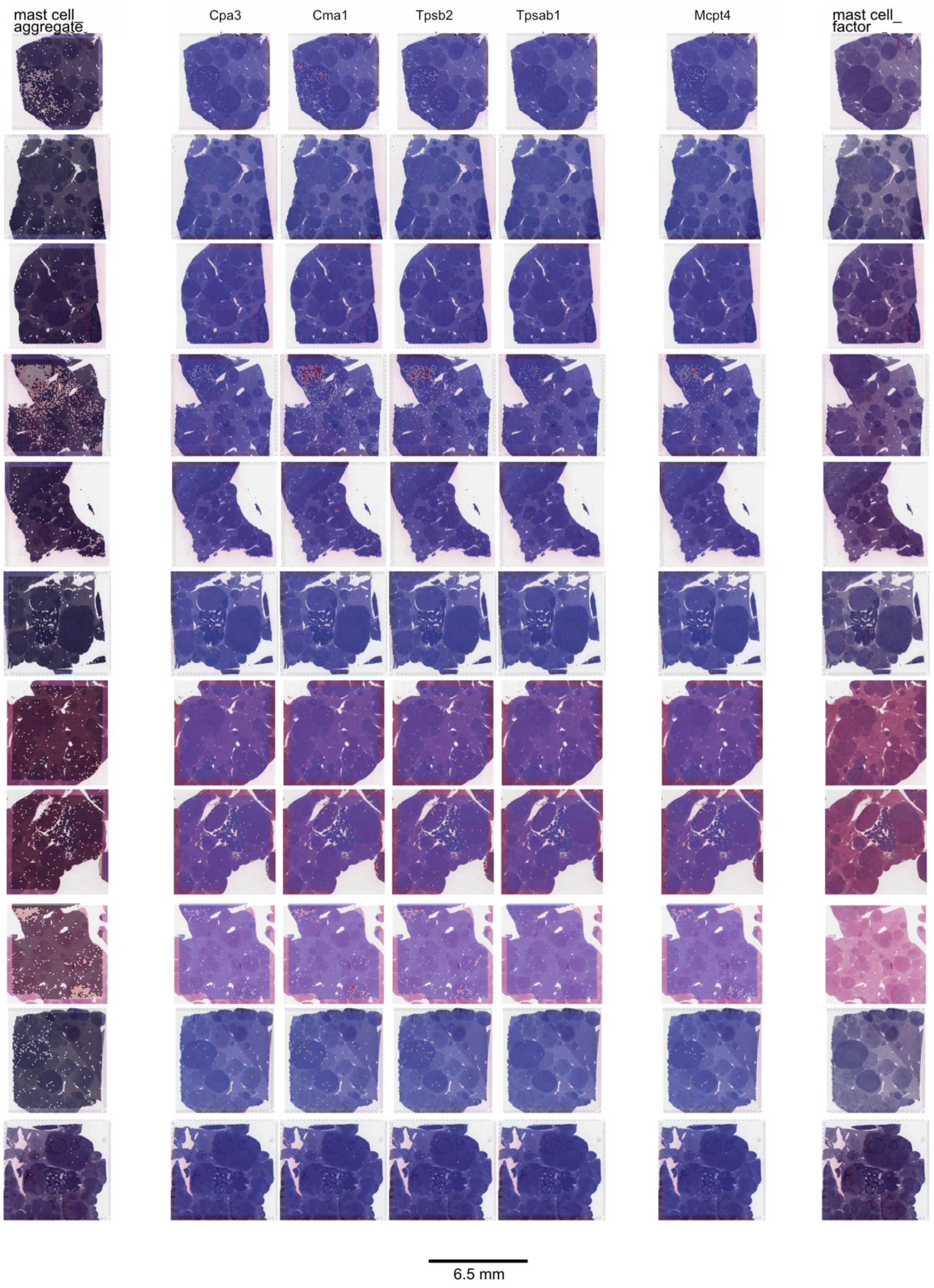

Supplementary Fig. 15 Mast cells-like phenotype. Aggregated phenotypes, expression of individual phenotype-associated transcripts, and factor loadings per phenotype of interest. Left-most column: Aggregated phenotype values per each phenotype. Right-most column: Estimated factor loadings per spot (Methods). Columns in between: Scaled expression of single “core markers”, as well as associated transcripts. As presented in Fig. 5. Data are depicted for all eleven 10X Visium samples used in this study. Samples are based on a single RUBIX experiment with 2 animals. See interactive web browser(https://chocolat-g2p.dkfz.de/).

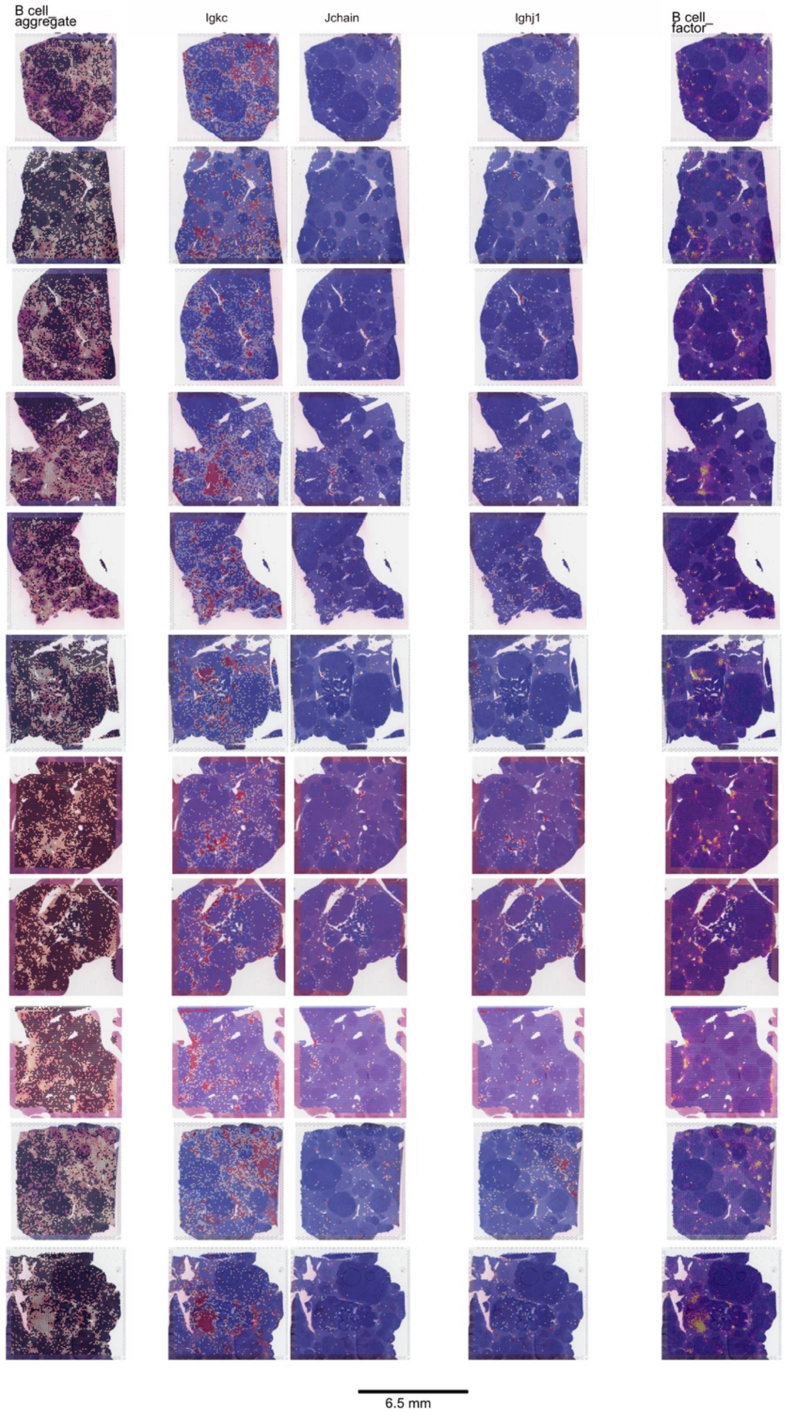

Supplementary Fig. 16: B-cell like phenotype. Aggregated phenotypes, expression of individual phenotype-associated transcripts, and factor loadings per phenotype of interest.Left-most column: Aggregated phenotype values per each phenotype. Right-most column: Estimated factor loadings per spot (Methods). Columns in between: Scaled expression of single "core markers", as well as associated transcripts. As presented in Fig. 5. Data are depicted for all eleven 10X Visium samples used in this study. Samples are based on a single RUBIX experiment with 2 animals. See interactive web browser(https://chocolat-g2p.dkfz.de/).

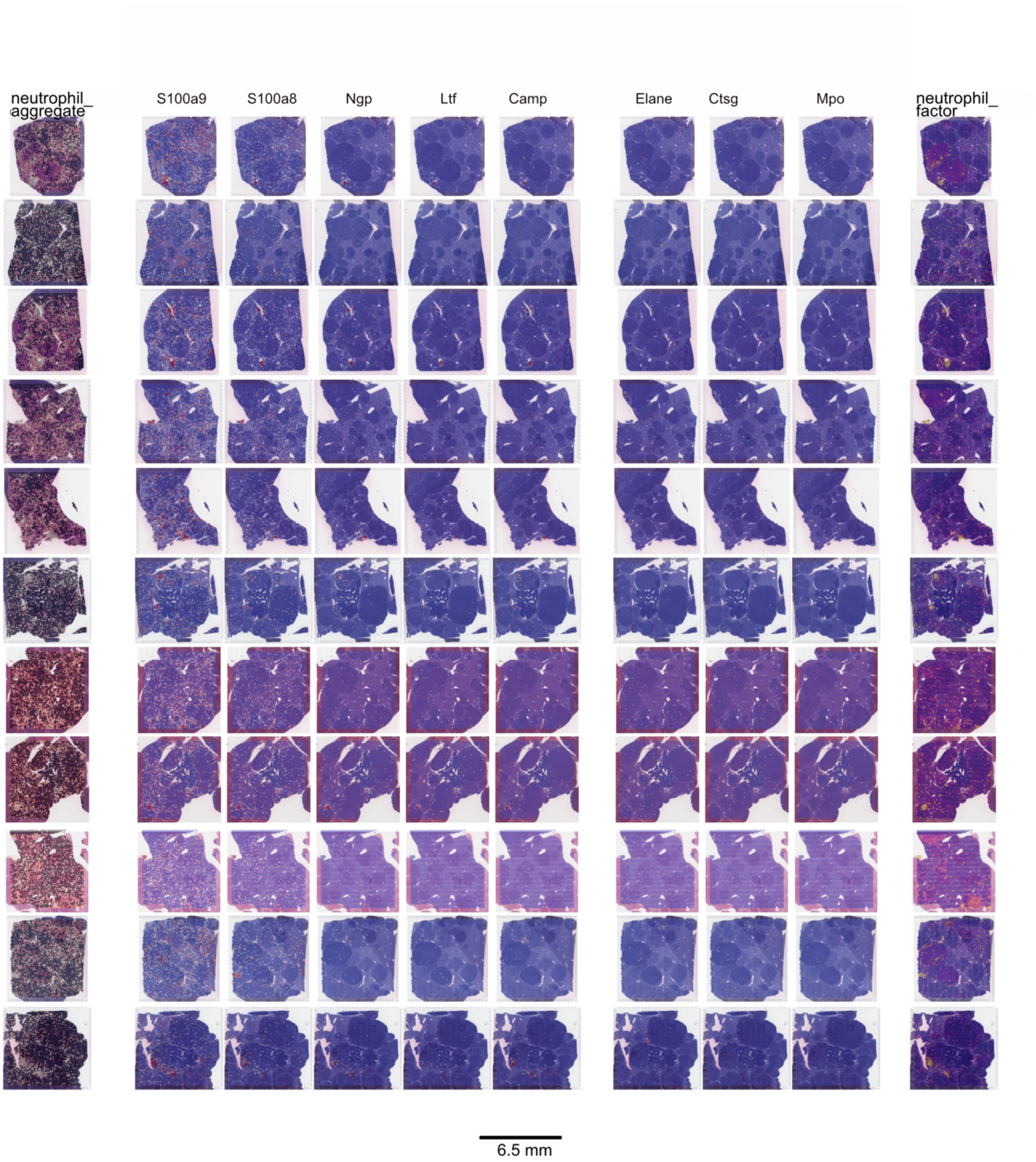

Supplementary Fig.17: Neutrophil-like phenotype. Aggregated phenotypes, expression of individual phenotype-associated transcripts, and factor loadings per phenotype of interest. Left-most column: Aggregated phenotype values per each phenotype. Right-most column: Estimated factor loadings per spot (Methods). Columns in between: Scaled expression of single "core markers", as well as associated transcripts. As presented in Fig. 5. Data are depicted for all eleven 10X Visium samples used in this study. Samples are based on a single RUBIX experiment with 2 animals. See interactive web browser(https://chocolat-g2p.dkfz.de/).

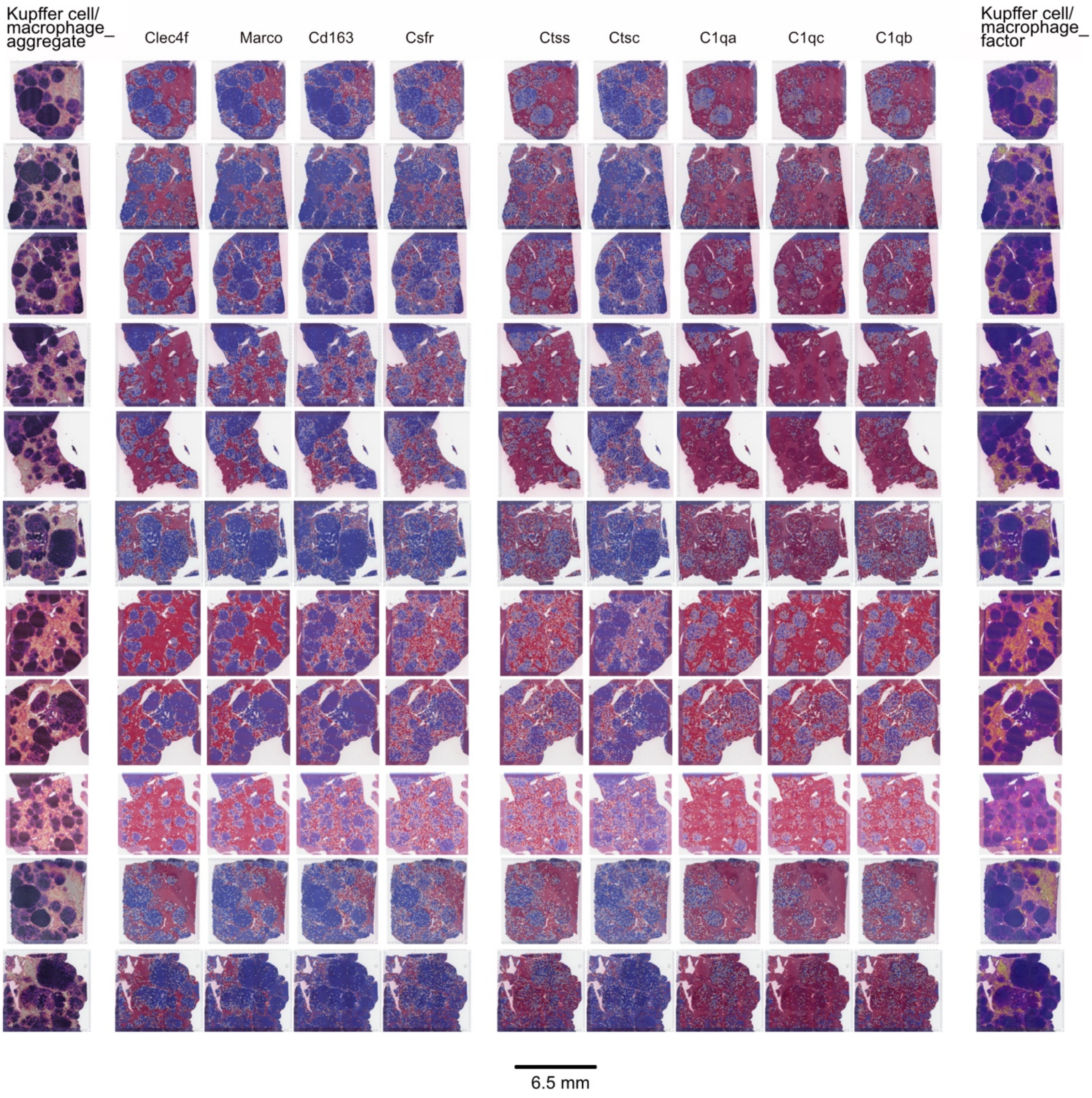

Supplementary Fig.18: Kupffer cell/macrophage-like phenotype. Aggregated phenotypes, expression of individual phenotype-associated transcripts, and factor loadings per phenotype of interest. Left-most column: Aggregated phenotype values per each phenotype. Right-most column: Estimated factor loadings per spot (Methods). Columns in between: Scaled expression of single “core markers”, as well as associated transcripts. As presented in Fig. 5. Data are depicted for all eleven 10X Visium samples used in this study. Samples are based on a single RUBIX experiment with 2 animals. See interactive web browser(https://chocolat-g2p.dkfz.de/).
