# Supplementary material for: Integrated in vivo combinatorial functional genomics and spatial transcriptomics of tumours to decode genotype-to-phenotype relationships
Source: Nat Biomed Eng. 2025 Jul 28;10(1):125–43. doi: 10.1038/s41551-025-01437-1 (PMC12823398; doi:10.1038/s41551-025-01437-1)
Supplement: Supplementary file 2 — Reporting Summary [file 41551_2025_1437_MOESM2_ESM.pdf]

nature portfolio

Corresponding author(s): Breinig Marco, Darjus Tschaharganeh, Moritz Gerstung

Last updated by author(s): Apr 11, 2025

Reporting Summary

Nature Portfolio wishes to improve the reproducibility of the work that we publish. This form provides structure for consistency and transparency in reporting. For further information on Nature Portfolio policies, see our Editorial Policies and the Editorial Policy Checklist.

Statistics

For all statistical analyses, confirm that the following items are present in the figure legend, table legend, main text, or Methods section.

n/a	Confirmed	
☐	☒	The exact sample size (*n*) for each experimental group/condition, given as a discrete number and unit of measurement
☐	☒	A statement on whether measurements were taken from distinct samples or whether the same sample was measured repeatedly
☐	☒	The statistical test(s) used AND whether they are one- or two-sided<br>*Only common tests should be described solely by name; describe more complex techniques in the Methods section.*
☐	☒	A description of all covariates tested
☐	☒	A description of any assumptions or corrections, such as tests of normality and adjustment for multiple comparisons
☐	☒	A full description of the statistical parameters including central tendency (e.g. means) or other basic estimates (e.g. regression coefficient) AND variation (e.g. standard deviation) or associated estimates of uncertainty (e.g. confidence intervals)
☐	☒	For null hypothesis testing, the test statistic (e.g. *F*, *t*, *r*) with confidence intervals, effect sizes, degrees of freedom and *P* value noted<br>*Give P values as exact values whenever suitable.*
☐	☒	For Bayesian analysis, information on the choice of priors and Markov chain Monte Carlo settings
☒	☐	For hierarchical and complex designs, identification of the appropriate level for tests and full reporting of outcomes
☐	☒	Estimates of effect sizes (e.g. Cohen's *d*, Pearson's *r*), indicating how they were calculated

Our web collection on statistics for biologists contains articles on many of the points above.

Software and code

Policy information about availability of computer code

Data collection	version of software that was used to get Visium 10x matrices (spaceranger 2.0.1.)
Data analysis	We used Python (v3.9.12) and the packages, anndata (v0.11), scanpy (v1.9.8), squidpy (v1.4.1), sagenet (v1.1.0), Cell2module (GitHub version, fetched 02.2024), pandas (v2.0.3), Torch (v2.1.1), Numpy (v1.24.3), Matplotlib (v3.7.2), Pyro (v1.8.6), SciPy (v1.11.3), and alpha_shape (GitHub clone c171a7d). We used R (v4.3.0) and the packages SingleCellExperiment (v1.24.0), ZellKonverter (v1.12.1), scater (v1.30.1), ComplexHeatmap (v2.16.0), FSA(0.9.6), dplyr(1.1.4), glasso (v1.11),ggplot2 (v3.5.1), igraph (v2.0.1.1), and scran (v1.28.2).<br><br>All scripts and custom code for data analysis are available at https://github.com/gerstung-lab/CHOCOLAT-G2P/.

For manuscripts utilizing custom algorithms or software that are central to the research but not yet described in published literature, software must be made available to editors and reviewers. We strongly encourage code deposition in a community repository (e.g. GitHub). See the Nature Portfolio guidelines for submitting code & software for further information.

Data

Policy information about availability of data

All manuscripts must include a data availability statement. This statement should provide the following information, where applicable:

- Accession codes, unique identifiers, or web links for publicly available datasets
- A description of any restrictions on data availability
- For clinical datasets or third party data, please ensure that the statement adheres to our policy

We used publicly available datasets from scLiverDB (https://guolab.wchscu.cn/liverdb#!/), PanglaoDB (https://panglaodb.se/), MSigDB (https://www.gsea-msigdb.org/gsea/msigdb), GEO (https://www.ncbi.nlm.nih.gov/geo/), MGI (https://www.informatics.jax.org/), and the LiverCellAtlas (https://www.livercellatlas.org/).
We deposited all data to https://zenodo.org/records/10986436/.
In addition, we have launched a web-browser for interactive data analyses (CHOCOLAT-G2P.dkfz.de).

Research involving human participants, their data, or biological material

Policy information about studies with human participants or human data. See also policy information about sex, gender (identity/presentation), and sexual orientation and race, ethnicity and racism.

Reporting on sex and gender	not applicable
Reporting on race, ethnicity, or other socially relevant groupings	not applicable
Population characteristics	not applicable
Recruitment	not applicable
Ethics oversight	not applicable

Note that full information on the approval of the study protocol must also be provided in the manuscript.

Field-specific reporting

Please select the one below that is the best fit for your research. If you are not sure, read the appropriate sections before making your selection.

☒ Life sciences ☐ Behavioural & social sciences ☐ Ecological, evolutionary & environmental sciences

For a reference copy of the document with all sections, see nature.com/documents/nr-reporting-summary-flat.pdf

Life sciences study design

All studies must disclose on these points even when the disclosure is negative.

Sample size	sample size for animal experiments was determined based on prior work: PMID: 29969439, 34509979
Data exclusions	We excluded one animal from the analysis due to failure of hydrodynamic tail vein injection for the initial experiment depicted in ED Fig.2a.
Replication	Corresponding hydrodynamic tail vein injection experiments were performed in n=2 animals per group for ST-based readouts.<br>Hydrodynamic tail vein injection experiments shown in Fig.7 were performed in n=4 animals per group.<br>For 10X Visium, we covered regions of interest via serial sections to provide replicas.<br>2 standard 10X Visium experiments were performed, one additional 10X CytAssist experiment was performed in which serial sections were included. All attempts at replication were successful.
Randomization	Animals were randomly assigned to the hydrodynamic tail vein injection experiments, ensuring unbiased allocation to experimental conditions. However, regions of interest (ROIs) for 10x Visium were not randomly selected; instead, they were chosen based on histopathological evaluation (H&E staining) to ensure the presence of at least 30 tumor nodules within a defined area (6.5 × 6.5 mm). This approach was necessary to ensure sufficient n of tumor nodules for analysis. Since this selection was based on objective histological criteria rather than experimental conditions, covariate bias was minimized.
Blinding	Blinding was not performed in this study because the selection of regions of interest (ROIs) for 10x Visium was based on objective histopathological evaluation (H&E staining). The requirement to observe at least 30 tumor nodules within a predefined area (6.5 × 6.5 mm) necessitated informed selection.

Reporting for specific materials, systems and methods

We require information from authors about some types of materials, experimental systems and methods used in many studies. Here, indicate whether each material, system or method listed is relevant to your study. If you are not sure if a list item applies to your research, read the appropriate section before selecting a response.

Materials & experimental systems

n/a	Involved in the study
☐	☒ Antibodies
☒	☐ Eukaryotic cell lines
☒	☐ Palaeontology and archaeology
☐	☒ Animals and other organisms
☒	☐ Clinical data
☒	☐ Dual use research of concern
☒	☐ Plants

Methods

n/a	Involved in the study
☒	☐ ChIP-seq
☒	☐ Flow cytometry
☒	☐ MRI-based neuroimaging

Antibodies

Antibodies used

CK19, 1:100, Abcam, Catalogue number: Ab133496
Hnf4alpha, 1:400, Abcam, Catalogue number: Ab181604
GS, 1:1000, BioScience, Catalogue number: BD610517
tRFP, 1:500, Evrogen, Catalogue number: AB233
GFP, 1:100, CellSignaling, Catalogue number: 2956

Validation

HNF4alpha, 1:400, Abcam, ab181604; Validation was performed by supplier.
https://www.abcam.com/en-us/products/primary-antibodies/hnf-4-alpha-antibody-epr16885-chip-grade-ab181604#
Specificity and sensitivity confirmed by provider in IHC with multi-tissue microarray (TMA) validation.

GS, 1:1000, BioScience BD610517; Validation was performed by supplier.
https://www.bdbiosciences.com/en-us/products/reagents/microscopy-imaging-reagents/immunofluorescence-reagents/purified-mouse-anti-glutamine-synthetase.610517?tab=product_details
Specificity and sensitivity confirmed by provider via Western blot analysis of glutamine synthetase on a rat cerebrum lysate and IHC of Glutamine synthetase staining on a rat cerebrum section.

tRFP. 1:500 Evrogen, AB233; Validation was performed by supplier.
https://evrogen.com/products/antibodies/AB-tRFP.shtml
Specificity and sensitivity confirmed by provider in multiple citations via expression of tRFP fusion constructs. https://evrogen.com/products/antibodies/AB-tRFP_Citations.shtml

GFP, 1:100, Cell Signaling 2956; Validation was performed by supplier.
https://www.cellsignal.com/products/primary-antibodies/gfp-d5-1-rabbit-mab/2956?srsltid=AfmBOorjtEQ-RhQgUyR-S9ekMndKszsrTErFlFyfqODMYaauPQUVgcce
Specificity and sensitivity confirmed by provider via Western Blot and IHC of PFA-embedded HCC827 cells transfected with GFP-plasmid.

CK19, 1:100 Abcam, ab133496;Validation was performed by supplier.
https://www.abcam.com/en-us/products/primary-antibodies/cytokeratin-19-antibody-epncir127b-ab133496?srsltid=AfmBOoqwF58K57WZlYYG0TaR6uEqXU3jJOMRTKhBS5F3i-0PWvL8dZab
Specificity and sensitivity confirmed by provider via IHC analysis of mouse colon tissue sections.

CK19, 1:100 Abcam, ab133496; Validation using our own data: Fig.7: CK19 staining is specifically observed in histophatologically well-defined cholangiocarcinoma nodules and normal bile duct cells as shown in Fig 7d.

Animals and other research organisms

Policy information about studies involving animals; ARRIVE guidelines recommended for reporting animal research, and Sex and Gender in Research

Laboratory animals

8-10 week old female C57Bl/6 animals were purchased from Envigo and used in this study. Housing conditions for the mice included a 12-hour light/12-hour dark cycle, an ambient temperature of 20–24°C, and relative humidity of 45–65%. All animal experiments were conducted in compliance with the regional regulations and in approval of the regional board Karlsruhe, Germany (G-81/20).

Wild animals	No wild animals were used in the study.
Reporting on sex	All mice in this study were female
Field-collected samples	No field collected samples were used in the study.
Ethics oversight	All animal experiments were approved by the regional board Karlsruhe, Germany (G-81/20)

Note that full information on the approval of the study protocol must also be provided in the manuscript.

Plants

Seed stocks	not applicable
Novel plant genotypes	not applicable
Authentication	not applicable
